# Intérêt du traitement chirurgical des fractures du massif trochantérien par clou gamma, à propos de 84 cas

**DOI:** 10.11604/pamj.2014.19.6.3190

**Published:** 2014-09-03

**Authors:** Jalal Boukhris, Mostapha Boussouga, Abdelouahab Jaafar, Belkacem Chagar

**Affiliations:** 1 Service de Traumatologie Orthopedie II- HMIMV, Rabat, Maroc

**Keywords:** Massif trochantérien, fracture, clou gamma, trochanteric mass, fracture, gamma nail

## Abstract

La fracture trochantérienne est une urgence différée qui se voit essentiellement chez le sujet âgé, dont les processus physiologique sont en déclin progressif. Chez le sujet jeune, elle est rare et souvent consécutive à un traumatisme violent. Ces fractures ont bénéficié de l’évolution constante des moyens et des techniques thérapeutiques, visant à améliorer l'ostéosynthèse, assurant ainsi un lever et un appui précoces. Nous rapportons une série de 84 cas de fractures du massif trochantérien traités chirurgicalement par clou gamma. Les résultats fonctionnels sont très encourageants en les comparants à ceux rapportés dans la littérature.

## Introduction

Les fractures du massif trochantérien représentent une urgence chirurgicale différée, devant être idéalement opérée dans les 48 heures. La fréquence de ces fractures ne cessera d'augmenter en raison du vieillissement de la population. Les moyens thérapeutiques, dont l'objectif est d'assurer un lever et une mise en charge précoces, ont nettement évolué, en l'occurrence les moyens de la chirurgie à ciel fermé. Le clou gamma représente l'un des derniers perfectionnements des implants destinés au traitement de ces fractures. Il s'agit d'un moyen de synthèse endomédullaire à foyer fermé.

## Méthodes

Les auteurs rapportent une étude rétrospective portant sur 84 patients opérés d'une fracture du massif trochantérien par un clou gamma colligées au service de Traumatologie-Orthopédie II de l'hôpital militaire d'instruction Mohamed V de Rabat sur une période de 5 ans, entre janvier 2008 et décembre 2012. Les méthodes d’étude ont été basées sur l'exploitation des dossiers médicaux avec recueil des données sur l'examen clinique, les données radiologiques, chirurgicaux et l’évolution chez ces patients.

## Résultats

**Figure 1:**
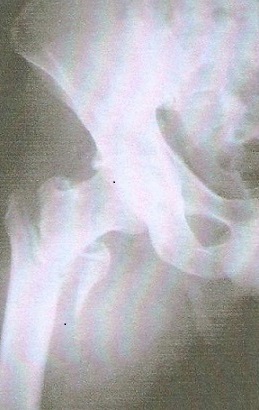
Radiographie de hanche droite de face montrant une fracture per-trochantérienne complexe du côté droit

**Figure 2:**
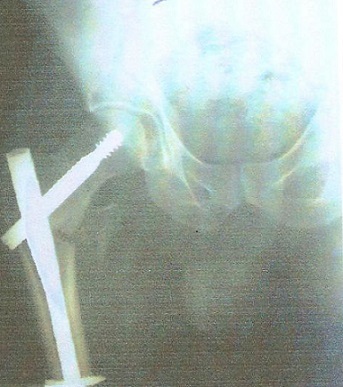
Contrôle radiologique post opératoire après ostéosynthèse par clou gamma

**Figure 3:**
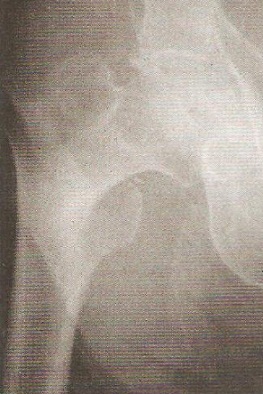
Fracture per-trochantérienne droite

**Figure 4:**
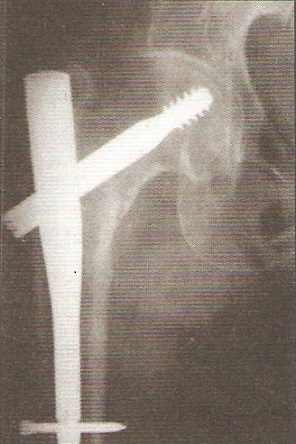
Contrôle radiologique post opératoire: ostéosynthèse par clou gamma

Le délai moyen de l'intervention était de 2 heures à 7 jours, ceci permet la mise en condition du patient. L'intervention a eu lieu 71 fois sous rachianesthésie, 4 fois sous anesthésie péri-durale et l'AG a été pratiquée chez le reste des patients. La durée moyenne de l'acte opératoire a été de 55 min avec des extrêmes de 40 à 90 min. Tous les clous utilisés dans notre série avaient une angulation de 130° avec un diamètre de 11 mm. Chez 9 patients on a eu recours à la version longue du clou gamma notamment en cas d'association avec des fractures diaphysaires homolatérales (Figure 1, Figure 2). La qualité de l'ostéosynthèse est jugée sur la position de la vis cervicale dans la tête, sur les clichés de face et de profil, considérant que la meilleure position était inférieure de face et médiane de profil venant soutenir les travées de compression primaires (Figure 3, Figure 4).

**Figure 5:**
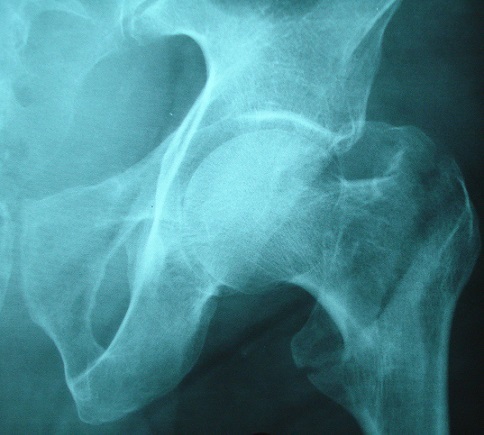
Fracture per-trochantérienne gauche

**Figure 6:**
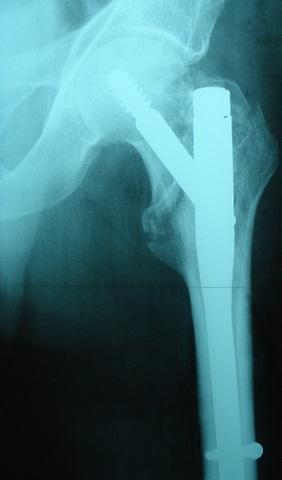
Contrôle radiologique post opératoire: ostéosynthèse par clou gamma

Au cours de l’évolution, 60 patients ont été mis en charge précocement durant les 10 premiers jours. Les complications postopératoires ont été de 3 types: complications septiques chez 3 patients jugulés par un traitement antibiotiques, une complication urinaire chez une seule patiente et un cas d'hématome postopératoire dont l’évolution était favorable sous drainage. La consolidation osseuse a été obtenue dans un délai de 3 à 6 mois (Figure 5, Figure 6). Le résultat fonctionnel a été jugé excellent et bons dans 82,5%, moyens et mauvais dans 17,5% des cas.

## Discussion

Les fractures du massif trochantérien, très fréquentes, sont l'apanage du sujet âgé en raison de l'ostéoporose et de l'atrophie musculaire mais peuvent se voir également chez le sujet jeune lors d'un traumatisme violent. L’âge moyen dans notre série est de 64 ans, c'est un âge relativement jeune. La majorité des séries font état d'une prédominance féminine qui va de 70 à 80% [1], dans notre série, nous avons constaté le phénomène inverse, ou l'homme a été atteint dans la proportion de 64,28% des cas, ceci est du au mode de recrutement de notre formation militaire. L'ostéosynthèse des fractures du massif trochantérien par le clou gamma, possède des avantages biomécaniques en réduisant le bras de levier au niveau du col fémoral par rapport à une plaque fixée sur la corticale latérale. Les forces de flexion sont considérablement réduites au profit des forces de compression au niveau du foyer de fracture tout en évitant la protrusion céphalique du matériel [2]. Ce système garantit un montage dynamique et stable à foyer fermé. Il est doté d'un ancillaire qui facilite la pose de l'implant; en effet malgré notre courte expérience, la durée moyenne de l'intervention a été relativement courte 55 min, celle-ci était de 41 min pour Kempf [3]. Le clou gamma présente les avantages de la chirurgie à foyer fermé. Il respecte l'hématome fracturaire, il évite l’ hémorragie et l'hématome lié à la désinsertion systématique du vaste externe dans l'ostéosynthèse juxta cortical. Par le même biais, le risque infectieux est très réduit [4]. Dans notre série, nous avons relevé trois cas d'infection superficielle d’évolution favorable.

La précocité de l'intervention est certainement un facteur déterminent de l’évolution du malade sauf contre indication médicale [5]. L'intervention ne devrait pas être différée de plus de 24 heures [6]. Dans la plupart des séries, le délai moyen d'intervention est à peine supérieur à 48 heures [7–9]. Dans notre série il est 2 fois plus élevé, ceci s'explique par le temps nécessaire à la préparation des patients à l'acte chirurgical, parfois long, sécurité indispensable en milieu hospitalier. Le lever précoce avec appui est fonction de la stabilité de la fracture, la qualité du montage et surtout de la qualité des structures osseuses du patient [10]. Dans la série de Kempf [3] qui comporte 69% de fracture instables, 83,4% ont eu un appui précoce. Chez nos malades, l'appui a été autorisé dès la première semaine chez 68 patients soit 81%. La consolidation a été obtenue chez 72 patients de notre série soit 85,71%. Kempf [3] a rapporté 99% de consolidation. Cette consolidation se fait, en général, entre le deuxième et le troisième mois, elle a été aux alentours de la dixième semaine dans 95% des cas.

## Conclusion

Le clou gamma, nouveau venu dans le traitement à foyer fermé par tuteur endo-osseux, représente le dernier perfectionnement du traitement des fractures trochantériennes selon le principe du clou verrouillé et du foyer fermé. Son utilisation dans le traitement des fractures du massif trochantérien, nous a donné plein satisfaction. La majorité des études biomécaniques sont en faveur de ces implants, qui permettent la mise en charge systématique et possèdent en plus les avantages de la chirurgie à ciel fermé.
